# Integrated model for genomic prediction under additive and non-additive genetic architecture

**DOI:** 10.3389/fpls.2022.1027558

**Published:** 2022-11-30

**Authors:** Neeraj Budhlakoti, Dwijesh Chandra Mishra, Sayanti Guha Majumdar, Anuj Kumar, Sudhir Srivastava, S. N. Rai, Anil Rai

**Affiliations:** ^1^ Division of Agricultural Bioinformatics, ICAR-Indian Agricultural Statistics Research Institute, New Delhi, India; ^2^ Department of Microbiology and Immunology, Dalhousie University, Halifax, NS, Canada; ^3^ Bioinformatics and Biostatistics Department, University of Louisville, Louisville, KY, United States

**Keywords:** GBLUP, GEBVs, k-RCV, nonparametric, parametric, SVM, RCV

## Abstract

Using data from genome-wide molecular markers, genomic selection procedures have proved useful for estimating breeding values and phenotypic prediction. The link between an individual genotype and phenotype has been modelled using a number of parametric methods to estimate individual breeding value. It has been observed that parametric methods perform satisfactorily only when the system under study has additive genetic architecture. To capture non-additive (dominance and epistasis) effects, nonparametric approaches have also been developed; however, they typically fall short of capturing additive effects. The idea behind this study is to select the most appropriate model from each parametric and nonparametric category and build an integrated model that can incorporate the best features of both models. It was observed from the results of the current study that GBLUP performed admirably under additive architecture, while SVM’s performance in non-additive architecture was found to be encouraging. A robust model for genomic prediction has been developed in light of these findings, which can handle both additive and epistatic effects simultaneously by minimizing their error variance. The developed integrated model has been assessed using standard evaluation measures like predictive ability and error variance.

## 1 Introduction

Genomic selection is a form of marker-assisted selection (MAS) in which genomic markers covering the whole genome are used to identify quantitative trait loci (QTL) which are in linkage disequilibrium (LD) with at least one marker (Meuwissen et al., 2001). Genomic selection predicts the breeding values of individuals or lines in a population by analyzing their phenotypes and high-density marker scores. The genomic selection process starts with building a statistical model from individuals having both genotypic and phenotypic information (i.e., training set); this model is further used for estimation of breeding value of the individuals in the breeding population/validation set (i.e., Genomic Estimated Breeding Value (GEBVs) for individuals having only genotypic information). Individuals are then ranked on the basis of GEBVs and subsequently superior individuals are selected. Genomic selection methods have been successfully applied for various plants (Jannink et al., 2010; Spindel et al., 2015; Zhao et al., 2015; Crossa et al., 2016; Liu et al., 2019) and animals (Hayes et al., 2009; Daetwyler et al., 2010; Daetwyler et al., 2012; Wang et al., 2013; Wolc et al., 2015; Lu et al., 2016; Wiggans et al., 2017; Liu et al., 2019), and reason behind this success is that it incorporates all information on genome wide markers into the prediction model.

As a choice of model, different methods that may be parametric, nonparametric, and semiparametric can be used for genomic selection. But, in general, it was observed that performance of parametric methods were considerably better than nonparametric methods in case of additive genetic architectures (Gianola et al., 2006; Crossa et al., 2010; Daetwyler et al., 2010; Heslot et al., 2012; Howard et al., 2014; Sahebalam et al., 2019). The practical use of genomic selection includes efforts such as appropriate statistical model selection, training and testing data proportions, marker density, etc., which requires resource-based decision-making. Prediction accuracy of a model can also be affected by factors like span of LD, heritability of trait under observation, and genetic architecture of individual under study. Due to the complexity of plant genetics, some genomic selection techniques perform very poorly as they are unable to model marker variance. Further, due to the huge number of epistatic interactions, it becomes challenging to practice parametric methods (Moore and Williams, 2009). In epistatic interactions, a number of loci are involved and also the possibility of interaction cannot be ignored. Epistatic interaction may play a crucial role for explaining genetic variation for quantitative traits, as ignoring these kinds of interaction in the model may result in lower genomic prediction accuracy (Gianola et al., 2006; Cooper et al., 2009). In such cases, performance of model free i.e. nonparametric methods were found to be more impressive (Gianola et al., 2006).

Although some semiparametric (Gianola et al., 2006; Campos et al., 2010; Legarra and Reverter, 2018) and other robust approaches (Tanaka, 2018; Budhlakoti et al., 2020a; Majumdar et al., 2020b; Sehgal et al., 2020; Mishra et al., 2021) have also been proposed and implemented for this purpose, there is still room for improvement. To overcome the limitation of individual parametric and nonparametric models, the current study has been designed to develop a robust model by integrating the best model from each category that can handle diverse genetic architecture.

## 2 Materials and method

In GS, our main objective is to select superior individuals by modelling the relationship between individual genotypic and phenotypic information. One of the simplest models for modeling this relationship is simple linear regression model. One problem with linear regression is that, generally, the number of markers (genotype) is greater than the number of individual (phenotype), that is, there exists a problem of large p and small n i.e., p > n. In such a case, it may not be possible to estimate parameters of regression model. Therefore, variable selection approach i.e., Ridge Regression (RR) and Least absolute Shrinkage and Selection Operator (LASSO), are alternatives to this situation. Some other improved methods include Best Linear Unbiased Prediction (BLUP) (Henderson, 1949), Genomic BLUP (GBLUP) (Endelman and Jannink, 2012), Bayesian methods, and their derivatives i.e. Bayes A, Bayes B, Bayes C *π* and D *π* (Meuwissen et al., 2001; Gianola et al., 2009; Habier et al., 2009; Habier et al., 2010). However, assumptions of parametric models do not always hold (e.g., normality, linearity, independent explanatory variables), which further suggests the use of nonparametric methods. Various nonparametric based methods, i.e. Reproducing Kernel Hilbert Space (RKHS), Support Vector Machine (SVM), Artificial Neural Network (ANN), and Random Forest (RF), have been proposed and successfully used for genomic prediction in plants and animals. A detailed comparison of various parametric and nonparametric methods has been provided by Howard et al., 2014; Budhlakoti et al., 2020b, in context to genomic selection.

### 2.1 Integrated estimation of GEBVs

The best model from each parametric and nonparametric methods was identified. Under parametric methods performance of GBLUP was found to be the best, whereas for nonparametric method, SVM was found to be best using appropriate evaluation measures. An integrated estimator for GEBVs (more formally GEBVs from parametric methods and EGV i.e. estimated genomic values from nonparametric methods) has been developed for genomic selection by combining estimates from the best parametric and nonparametric methods (Majumdar et al., 2020a and Majumdar et al., 2020b). For better understanding, details of both the methods have been given below.

### 2.2 Best linear unbiased prediction

BLUP is based on the theory of mixed random effect model. Statistical formulation of the BLUP model can be written as follows:


Y=Xβ+Zm+e

where, β is a p × 1 vector of fixed effects, m is q × 1 vector of random effects, m~N (0,G)   and e is n × 1vector of residuals, e ~N (0, R).   The estimator of fixed effect β is called Best Linear Unbiased Estimator (BLUE) and random effects m is known as BLUP. Estimation of BLUE and BLUP (*β, m*) by maximizing the joint likelihood function is given below (Henderson, 1949):


f(Y,m )= f(Y|m)f(m)

$$=\frac{1}{2\pi^{n/2}{\left|R\right|}^{1/2}}\left[-\frac{1}{2}(Y-X\beta -Zm)'R^{-1}(Y-X\beta -Zm)\right]\times \frac{1}{2\pi^{p/2}{\left|G\right|}^{1/2}}\left[-\frac{1}{2}m'G^{-1}m\right]$$

The estimate of (β, m) could be obtained by maximizing the log of the above likelihood function and equating it to zero, which could be written as the famous Henderson mixed model equation:


$$\left(\begin{array}{cc} X^{'}R^{-1}X & X^{'}R^{-1}Z\\ Z^{'}R^{-1}X & Z^{'}R^{-1}Z+G^{-1} \end{array}\right)\left(\begin{array}{c} \hat{\beta }\\ \hat{m} \end{array}\right)=\left(\begin{array}{c} X^{'}R^{-1}Y\\ Z^{'}R^{-1}Y \end{array}\right)$$

where G = var (m) and R = var (e). The solution to the Henderson equation is BLUE of β, BLUP of m, where m and e are normally distributed and maximizes f (Y, m) over unknown parameters β and m.

GBLUP is an improved version of BLUP where additive genomic relationship matrix (G) is used as a variance-covariance matrix of random effect in the model.

### 2.3 Support vector machine

SVM is based on the principle of maximum separating hyperplane. It constructs a hyperplane with the objective of separating data into different classes. In case our problem is based on regression instead of classification, i.e., when output data is continuous in nature, then the Support Vector Regression can be used. Support Vector Regression (SVR) is an important application of SVM technique and has been used interchangeably in the literature. In order to understand this, consider a mapping function *f (X*): *R^p^* →*R*, given the set of training data


$$(X_{1},Y_{1}),(X_{2},Y_{2})\;,\;.\;.\;,(X_{n},Y_{n}),\;X_{i}\in R^{p},\;Y_{i}\in R$$

Let us assume a simple linear function of the following form:

f (X) = w’X + b, where,  *w*  is vector of weight to be estimated (i.e. regression coefficients) and b denotes bias. f (X) is minimized by the following problem formulation:


$$\min_{w,b}\emptyset (w,b)=\frac{1}{2}{\left||w|\right|}^{2}+c\sum_{i=1}^{n}e_{i}^{k}$$

where e_i_ = Y_i_ – f (X_i_), is error of i^th^ data point from training set, also known as loss function L(.) which measures quality of estimation, and c represents regularization parameter which handles trade-off between margin and error.

### 2.4 Proposed estimator

The integrated estimator for estimated breeding or genomic value can be expressed as


(1) $$Y_{Est}=wY_{GBLUP}+\text{ (}1\;–w)Y_{SVR}$$

where, *Y_Est_* is new predicted phenotype from integrated model, w is $\frac{\sigma_{SVR}^{2}}{\sigma_{GBLUP}^{2}+\sigma_{SVR}^{2}}$ , where $\sigma_{SVR}^{2}$ and $\sigma_{GBLUP}^{2}$ are the error variance of models SVR and GBLUP respectively, Y_GBLUP_ is the predicted GEBV from GBLUP, whereas Y_SVR_ is the predicted EGV from SVR model. Let us assume that error variance of Y_Est_ is represented by $\sigma_{EST}^{2}$ , then by optimizing w, $\sigma_{EST}^{2}$ can be obtained as:


(2) $$\begin{array}{l} \sigma_{Est}^{2}={\left(\frac{\sigma_{SVR}^{2}}{\sigma_{GBLUP}^{2}+\sigma_{SVR}^{2}}\right)}^{2}\sigma_{GBLUP}^{2}+{\left(\frac{\sigma_{GBLUP}^{2}}{\sigma_{GBLUP}^{2}+\sigma_{SVR}^{2}}\right)}^{2}\sigma_{SVR}^{2}\\ \sigma_{Est}^{2}=\frac{\sigma_{GBLUP}^{2}\sigma_{SVR}^{2}}{\sigma_{GBLUP}^{2}+\sigma_{SVR}^{2}} \end{array}$$

### 2.5 Estimation of error variance for proposed estimator

**Figure 1:**
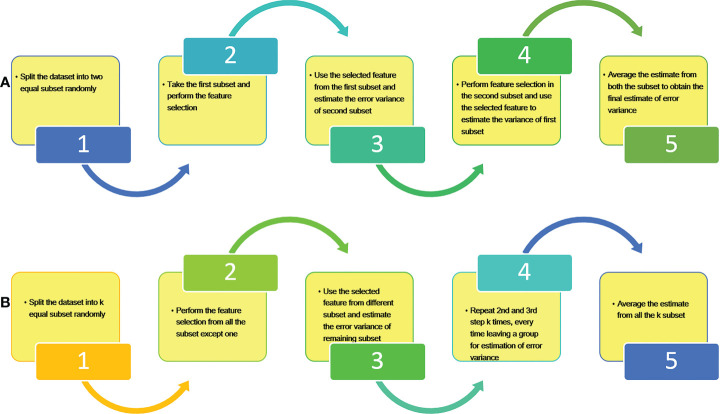
Basic steps for estimation of error variance using **(A)** RCV and **(B)** k-RCV.

In order to develop the integrated genomic selection model, estimate of error variances for GBLUP $\left(\sigma_{GBLUP}^{2}\right)$ and SVR $\left(\sigma_{SVR}^{2}\right)$ models have been obtained using two different methods i.e. Refitted Cross Validation (RCV) and k fold Refitted Cross Validation (k-RCV). RCV method was originally given by Fan et al., 2012, for the estimation of error variance in ultrahigh dimensional regression procedure. The basic procedure behind RCV and k-RCV is the same except that data is split into two equal halves for RCV and k equal sizes for k-RCV respectively. Algorithm of both RCV and k-RCV methods are depicted through the flow diagrams in **Figure 1**.

### 2.6 Data simulation

In order to check the performance of the model, data was simulated using QTL Bayesian interval mapping method implemented in R based package “*qtlbim*” (Yandell et al., 2007). R is open source and freely available at http://www.r-project.org (R Core Team, 2019). Package “*qtlbim*” is based on Cockerham’s model which is a standard model for simulation of marker data and has been followed in many studies (Bedo et al., 2008; Piao et al., 2011; Howard et al., 2014; Budhlakoti et al., 2020a; Budhlakoti et al., 2020b; Li et al., 2020).

Statistical formulation of Cockerham’s model is given as follows:


$$Y_{ijk}=G_{ij}+e_{ijk}$$

(3) $$=\mu +a_{1}x_{1}+d_{1}z_{1}+a_{2}x_{2}+d_{2}z_{2}+i_{aa}w_{aa}+i_{ad}w_{ad}+i_{da}w_{da}+i_{dd}w_{dd}+e_{ijk}$$

where *μ* is the mean, *a*_1_ and *a*_2_ are additive genetic effects at locus A & B, *d*_1_ and *d*_2_ are dominance effects at locus A & B, *e*_ijk_ is a residual. *i_aa_* is additive × additive effect of loci A and B, *i_ad_* is additive × dominance of loci A and B, *i_da_* is dominance × additive of loci A and B, and *i_dd_* is dominance × dominance of loci A and B.

We have simulated a total of five data sets for genotypic and phenotypic information using the Cockerham’s model described above (Eq. 3) with diversified genetic architecture (additive and epistasis) at various levels of heritability (ranges from low heritability 0.3 to medium 0.5 and high heritability 0.7 for F2 population). For the additive data, there is one QTL in each chromosome with either a positive or negative additive effect and no epistatic interaction say it as (*a, e*_0_). For non-additive/epistatic data, we assumed two QTLs on each of the five, seven, and ten chromosomes respectively; remaining chromosomes have no QTL. So, a total 5, 7, and 10 two-way epistatic interactions are considered for the non-additive datasets. So in each dataset, there is a combination of one of the five different levels of heritability (viz. 0.3, 0.5, 0.7) and four levels of epistatic effects (viz. 0, 5, 7, 10) denoted as *e*_0_, *e*_1_, *e*_2_, *e*_3_. So, finally, we have four ifferent combinations of datasets with additive and epistatic effects i.e. (*a, e*_0_), (*a, e*_1_), (*a, e*_2_) and (*a, e*_3_). For each genetic architecture we have simulated the data for 200 individuals with 2000 SNPs each. Simulated data have 10 chromosomes with 200 SNPs in each with specified length. A total of 2000 markers are distributed over all 10 chromosomes in such a way that each marker is equi-spaced over the chromosome. No missing genotypic values and no missing phenotypic values are considered in the datasets.

### 2.7 Real data set

In order to check the robustness of our approach the same has been validated using real data. We have used a total of six datasets in the current study. A detailed discussion regarding each of the dataset is given below.

#### 2.7.1 Dataset 1: Wheat

Wheat lines were genotyped using 1447 Diversity Array Technology markers generated by Triticarte Pty. Ltd. (Canberra, Australia; http://www.triticarte.com.au). Markers are coded for two different values i.e. their presence (1) or absence (0). This data set includes 599 lines phenotyped for trait grain yield (GY) for four mega environments. However, for matter of convenience we have just considered GY for the first mega environment. The final number of DArT markers after quality control and final editing was 1279 and the same was used in the current study (Crossa et al., 2010; Cuevas et al., 2016).

#### 2.7.2 Dataset 2: Maize

The maize dataset is generated by CIMMYT’s Global Maize Program (Crossa et al., 2010). It originally included 300 maize line with 1148 SNP markers. Markers with the highest frequency are coded as 0 and lowest frequency as 1. Here also the trait under study is GY, evaluated under drought and watered conditions. After final editing, 264 maize lines with 1135 SNPs markers were available for final study (Crossa et al., 2010).

#### 2.7.3 Dataset 3-6: Wheat

This wheat dataset is generated from CIMMYT semiarid wheat breeding program, which is comprised of 254 advanced wheat breeding lines genotyped for 1726 DArt markers (Poland et al., 2012). Dataset is recorded for four different phenotypic traits: Days to Heading (DTH), Thousand Kernel Weight (TKW), Yield (under irrigated condition hence denoted as Y_I_), and Yield (under draught condition i.e. Y_D_). For convenience, here trait DTH is considered as Dataset-3, trait TKW as Dataset-4, trait Y_I_ as Dataset-5, and trait Y_D_ as Dataset-6.

### 2.8 Evaluation measure

Predictive Ability and Prediction Error were used for evaluation of the different models. Predictive ability can be defined as Pearson correlation coefficient (r) between observed phenotypic value and predicted phenotypic value. The same can be expressed as (Eq. 4)


(4) $$r=\frac{S_{Y,\hat{Y}}}{S_{Y}S_{\hat{Y}}}$$

where $S_{Y,\hat{Y}}$ denotes the covariance between observed and predicted phenotypic value, *S_Y_* is standard deviation of observed phenotype, and $S_{\hat{Y}}$ denotes standard deviation of predicted phenotype. Prediction error can be simply defined as mean sum of square error (MSE) between observed phenotypic value and predicted phenotypic value. The same can be expressed using the following formula (Eq. 5)


(5) $$MSE=\frac{1}{n}\sum_{i=1}^{n}{\left(Y_{i}-{\hat{Y}}_{i}\right)}^{2}$$

where *Y_i_* is observed response, ${\hat{Y}}_{i}$ is predicted phenotype value of i^th^ individual, and *n* denotes total number of individuals in the training set.

**Figure 2:**
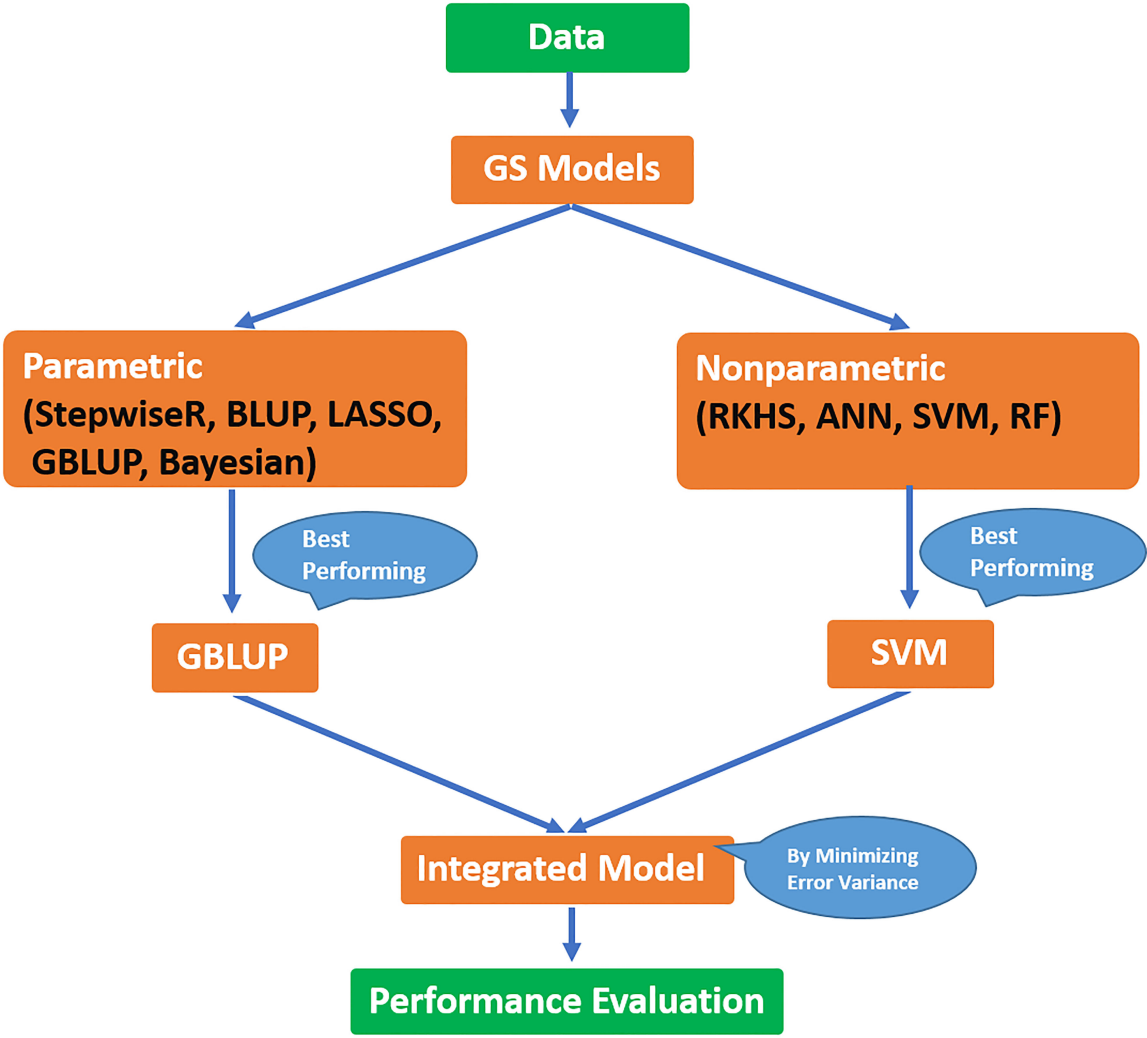
Flow diagram for the procedure followed to develop the integrated model in the current study.

To compare the performance of methods under study, a cross-validation technique is used. Data is divided into two parts, i.e., training and validation sets, in such a way that the training set comprises 70% of data and the rest of the data is in the validation set. The former is used for model building and the latter for model evaluation. The whole procedure is repeated 100 times and predictive ability and prediction error were calculated. For better understanding, a brief flowchart of the whole procedure followed in the current study is provided in **Figure 2**.

In order to implement all the methods under study, R programming platform (R Core Team (2019). R: A language and environment for statistical computing, R foundation for statistical computing Vienna - Google Search) was used; to fit different models under study, R package STGS was used (Budhlakoti et al., 2019).

## 3 Results and discussion

### 3.1 Comparative study of existing parametric methods

Here, using a simulation analysis, the most popular methods (i.e., Stepwise Regression, BLUP, LASSO, Bayesian LASSO, and GBLUP) for genomic selection under diverse genetic architectures were examined. Each method was evaluated at different heritability levels (i.e. 0.3, 0.5, and 0.7). Cross-validation technique was used to assess the performance of various models, and results of the same are presented in **Table 1**.

**Table 1:** Predictive ability and MSE of GEBVs for different parametric methods using simulated dataset at different levels of heritability (h^2^).

h^2^/Parameters		GBLUP	BayseianLasso	StepwiseR	BLUP	LASSO
**0.3**	**PA**	0.74	0.72	0.52	0.70	0.72
	**MSE**	0.26	0.18	0.92	0.26	0.21
**0.5**	**PA**	0.86	0.83	0.42	0.84	0.83
	**MSE**	0.23	0.25	0.90	0.24	0.23
**0.7**	**PA**	0.89	0.86	0.48	0.87	0.86
	**MSE**	0.32	0.32	0.88	0.32	0.24

The following critical observations can be made from the results (**Table 1**).

- At low heritability (0.3), the performance of GBLUP was found to be the highest and reasonable. However, performance of BLUP and Bayesian LASSO were also quite impressive. It can also be observed that as heritability increases, the performance of LASSO in comparison to other methods quickly improves.
- At moderate heritability (0.5), performance of GBLUP is highest in comparison to the others. However, an important thing to note is that there is not much difference in the performance of all the methods except stepwise regression.
- At high heritability (0.7), consistency in the performance of GBLUP is still maintained, with the performance of other methods (BLUP, LASSO, and Bayesian LASSO) also at par with GBLUP.
- Performance of stepwise regression is very low throughout at all levels of heritability. This makes this method unsuitable for genomic selection studies.
- LASSO can also be used as one of the preferable statistical models for genomic selection studies, especially when additive effects are present, but only for high heritable traits.
- For real-time scenarios (e.g., agriculture field data) where trait heritability is generally low (for most commonly studied yield related traits), GBLUP can be quite good for genomic selection studies. Results indicates that GBLUP has better predictive ability of estimating GEBVs of individuals over their counterparts.

### 3.2 Comparative study of existing nonparametric methods

This section summarizes the performance of different nonparametric methods under study, i.e., RKHS, SVR, ANN, and RF, at diverse levels of heritability. Predictive ability and prediction error were used as evaluation measures for different models. Results of the same are presented in **Table 2**.

**Table 2:** Predictive ability and MSE of EGVs for different nonparametric methods under study using simulated dataset for various levels of heritability (h^2^).

h^2^/Parameters		RKHS	ANN	SVR	RF
**0.3**	**PA**	0.53	0.72	0.75	0.63
	**MSE**	0.67	0.60	0.47	0.78
**0.5**	**PA**	0.55	0.82	0.85	0.70
	**MSE**	0.86	0.55	0.55	0.60
**0.7**	**PA**	0.62	0.84	0.88	0.72
	**MSE**	0.93	0.58	0.54	0.71

On the basis of results obtained (**Table 2**), the following inferences can be drawn:

- Performance of SVR was consistent throughout different levels of heritability with respect to its predictive ability and MSE.
- However, ANN also performed quite well, almost at par with SVR. Performance of random forest was poor at low heritability, however it improved gradually with high heritability.
- Performance of RKHS and RF were not found to be encouraging in comparison to their counterparts throughout the study.

From the above discussion, two models, GBLUP and SVR, each from parametric and nonparametric respectively, can been considered as the best model based on their performances in terms of estimating GEBVs and EGVs respectively for selection of individuals. Using these results, a robust model has been developed by integrating GBLUP and SVR by minimizing their error variance. Detailed results regarding error variance estimated using different methods is given below.

### 3.3 Comparison of error variances for GBLUP, SVM and integrated model

Here, two different methods for estimation of error variances, i.e., RCV and k-RCV, have been used for GBLUP, SVR, and Integrated model. Results of the same have been presented one by one in the tables given below.

#### 3.3.1 Refitted cross validation

Error variance estimated using Refitted Cross Validation (RCV) for GBLUP, SVR, and Integrated model is presented in **Table 3**.

**Table 3:** Error variance for different GS models at different heritability using RCV.

h^2^	GBLUP	SVR	Integrated Model
**0.3**	1.12	4.57	0.90
**0.5**	0.94	9.39	0.85
**0.7**	1.10	22.84	1.05

From **Table 3**, it has been observed that error variance of the integrated model is less than the error variance of GBLUP and SVR at diverse genetic architectures i.e., irrespective of levels of heritability and genetic effects.

#### 3.3.2 k-fold refitted cross validation

Error variances estimated using k-fold refitted cross validation (i.e., k-RCV) for GBLUP, SVR, and Integrated model were given in **Table 4**.

**Table 4:** Error variance for different GS models at different heritability using k-RCV.

h^2^	GBLUP	SVR	Integrated Model
**0.3**	1.04	4.57	0.85
**0.5**	1.05	9.91	0.95
**0.7**	1.28	26.72	1.22

From **Table 4**, it has also been observed that the error variance of the integrated model was found to be less than GBLUP and SVR across all levels of heritability using k-RCV approach.

**Figure 3:**
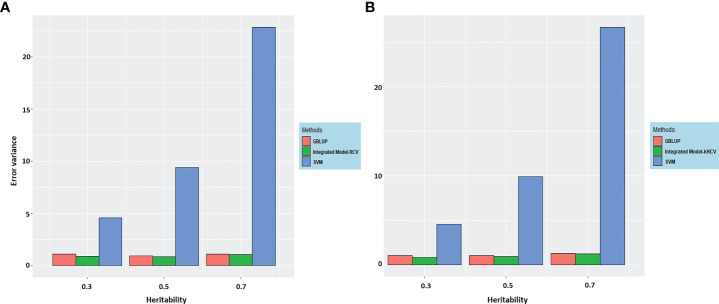
Error variance for integrated GS model at different heritability using various methods **(A)** RCV and **(B)** k-RCV in comparison to the error variance of best methods from both parametric and nonparametric i.e. GBLUP and SVR (Results from **Tables 3**, **4**).

In order to compare and better understand the results obtained through different methods of estimations for error variance (i.e., RCV, k-RCV), the same has been presented graphically in **Figure 3**.

The following important findings can be drawn from the results (**Figure 3**).

- The error variance estimated through RCV and k-RCV is almost similar. However, variance estimated through RCV is slightly lower than k-RCV; this difference may be caused by the reduced sample size in case of k-RCV.
- Our proposed method is robust to both architecture (i.e., additive and epistatic) as evidenced from error variance obtained through RCV and k-RCV.
- Error variance obtained through RCV and k-RCV is highest for SVR in comparison to BLUP and the integrated model.
- In general, error variance increases with increase in heritability level across the various methods.

### 3.4 Performance of error variance estimation methods for integrated model

Here we have presented the results of different error variance estimation methods (RCV and k-RCV) in terms of their capability and how accurately it gives GEBVs or EGVs. The same has been calculated using each approach, i.e., GBLUP, SVR, and integrated model, and the predictive ability of each of them was observed.

#### 3.4.1 Refitted cross validation

Predictive ability for GBLUP, SVR, and the integrated model using RCV variance is given below in the table at different levels of heritability and genetic effect.

#### 3.4.2 k-fold refitted cross validation

Predictive ability for GBLUP, SVR, and the integrated model using k-RCV variance is given below in the table at different levels of heritability and genetic effect.

The following important findings can be drawn from the results obtained in **Tables 5**, **6**:

- Performance of GBLUP is good when data have only additive architecture, while SVR performs equally well with diverse genetic architecture (with and without epistasis), especially at low heritability.
- At low heritability, the performance of the integrated model is consistent and robust.
- However, at high heritability (i.e. h^2 =^ 0.5 & 0.7), the performance of all the models in terms of prediction accuracy are at par.
- With increasing levels of epistasis and heritability, the predictive ability of the integrated model is still maintained

**Table 5:** Predictive ability (PA) with its standard error (SE) for different genomic selection models on mixed architecture (additive and epistatic effects) using RCV variance.

h^2^		GBLUP(*a*,*e*_0_)	GBLUP(*a*,*e*_1_)	SVR(*a*,*e*_1_)	SVR(*a*,*e*_0_)	Integrated Model(*a*,*e*_0_)	Integrated Model(*a*,*e*_1_)	Integrated Model(*a*,*e*_2_)	Integrated Model(*a*,*e*_3_)
**0.3**	**PA**	0.74	0.68	0.75	0.74	0.75	0.74	0.70	0.64
	**SE(PA)**	0.045	0.072	0.062	0.063	0.059	0.056	0.055	0.06
**0.5**	**PA**	0.86	0.84	0.85	0.81	0.88	0.85	0.82	0.79
	**SE(PA)**	0.032	0.045	0.041	0.048	0.035	0.027	0.03	0.03
**0.7**	**PA**	0.89	0.87	0.88	0.85	0.90	0.89	0.84	0.81
	**SE(PA)**	0.030	0.039	0.032	0.041	0.024	0.020	0.02	0.02

**Table 6:** Predictive ability (PA) with its standard error (SE) for different GS models at different heritability using k-RCV variance.

h^2^		GBLUP(*a*,*e*_0_)	GBLUP(*a*,*e*_1_)	SVR(*a*,*e*_1_)	SVR(*a*,*e*_0_)	Integrated Model(*a*,*e*_0_)	Integrated Model(*a*,*e*_1_)	Integrated Model(*a*,*e*_2_)	Integrated Model(*a*,*e*_3_)
**0.3**	**PA**	0.74	0.68	0.75	0.74	0.76	0.74	0.70	0.66
	**SE(PA)**	0.045	0.072	0.062	0.063	0.048	0.045	0.04	0.042
**0.5**	**PA**	0.86	0.84	0.85	0.81	0.88	0.87	0.82	0.80
	**SE(PA)**	0.032	0.045	0.041	0.048	0.029	0.026	0.032	0.041
**0.7**	**PA**	0.89	0.87	0.88	0.85	0.91	0.89	0.85	0.82
	**SE(PA)**	0.030	0.039	0.032	0.041	0.027	0.024	0.032	0.03

**Figure 4:**
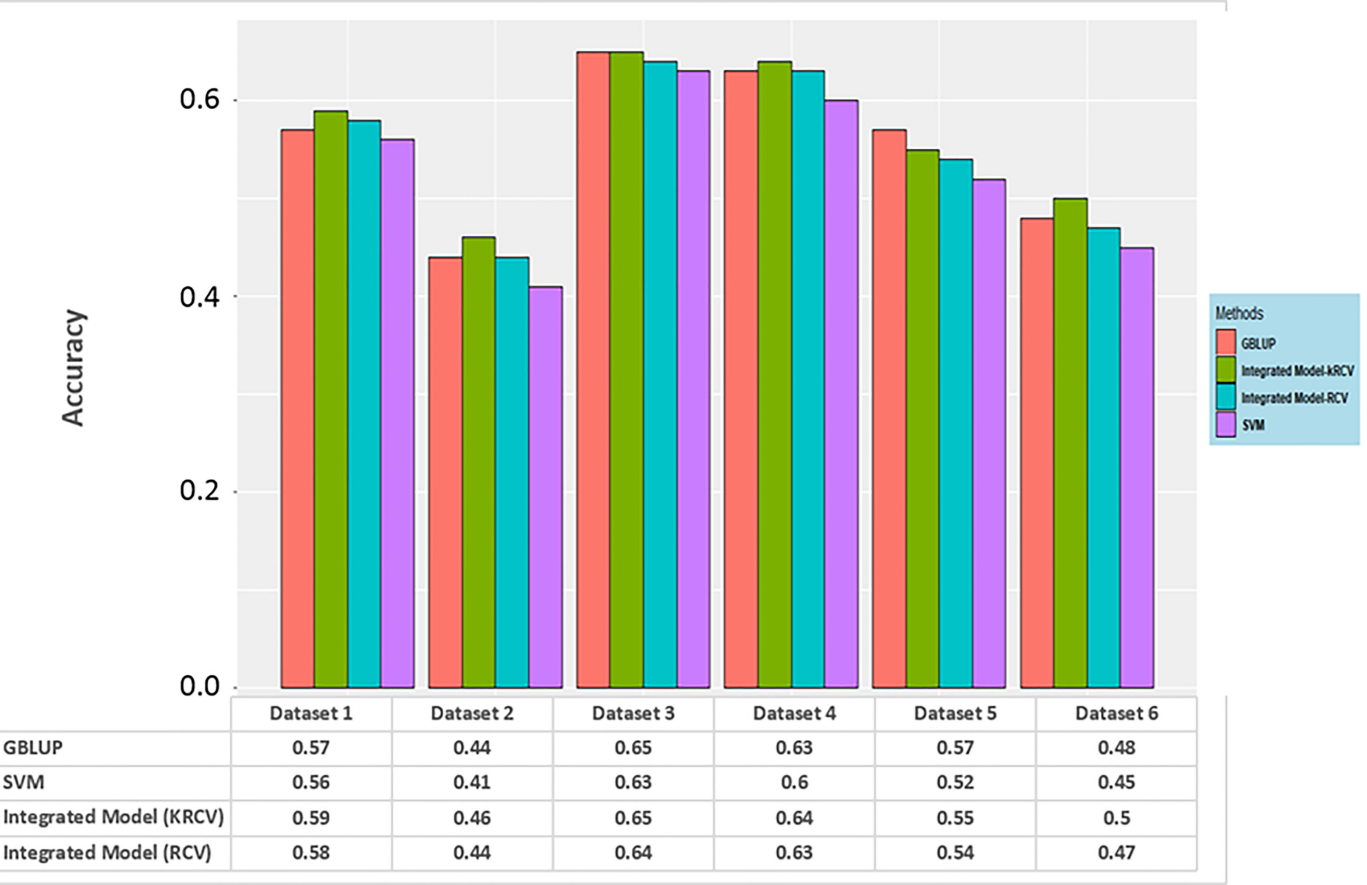
Prediction accuracy for different genomic selection models on a real dataset.

In order to support the facts obtained from the results of the simulation study, the same has also been tested on real datasets. Results obtained from the real dataset also tells the same story; here prediction accuracy for the integrated model is either at par or better than GBLUP and SVR model. However, here also the performance of k-RCV is slightly better than RCV. Graphical representation of the same is given below (**Figure 4**).

From the above discussion, two models, GBLUP and SVR, each of parametric and nonparametric respectively, can be considered the best models based on their performance in terms of reduced error variance and improved estimation of GEBVs and EGVs, respectively, for the selection of individuals. On the basis of this result, a robust model has been developed in this study by integrating GBLUP and SVR based on suitable weightage according to their error variance.

### 3.5 Practical deployment to the breeding programs

Here we present the R script as supplementary information for estimating the GEBVs of an individual using the integrated model (Supplementary File S1). The user may also run different GS-based models using a variety of other publicly accessible R tools & packages. In the future, GS-based tools or R packages may be developed that incorporate advanced and other GS-based models for hassle-free implementation.

## 4 Conclusion

In the current study, an effort has been made to develop a comprehensive methodology that addresses both the advantages and disadvantages of each parametric and nonparametric model. The performance of the GBLUP and SVR models was determined to be the best among its counterparts for both the parametric and nonparametric frameworks, respectively. The predictive ability and error variance of the developed integrated model were assessed, and it was found that our proposed approach performs either better or at par with existing models. It has also been observed that our proposed model is good at handling the diverse genetic architecture, i.e., additive and epistatic, in terms of reducing the error variance and enhancing the predictive ability. As a future directive, developed methodology could be evaluated by measuring the impact of within and across family predictive ability and other cross validation schemes.

## Data availability statement

Publicly available datasets were analyzed in this study. This data can be found here: Crossa et al., 2010; Cuevas et al., 2016.

## Author contributions

Conceived and designed the study: AR, NB, and DM. Developed the methodology: DM and NB. Performed the experiments: NB. Analyzed the data: NB and SM. Contributed materials: NB, DM, SS, SM, and AK. Drafted the manuscript: NB. Corrected the manuscript: AR, DM, NB, SS, and SR. All authors contributed to the article and approved the submitted version.

## Funding

The funding support received from ICAR sponsored CABin scheme network project entitled “Agricultural Bioinformatics and Computational Biology”.

## Acknowledgments

The authors sincerely acknowledge the fellowship support received from PG School ICAR-IARI and ICAR-IASRI to conduct this research and analysis. Authors duly acknowledge the computational support received from Advanced Supercomputing Hub for OMICS Knowledge in Agriculture (ASHOKA).

## Conflict of interest

The authors declare that the research was conducted in the absence of any commercial or financial relationships that could be construed as a potential conflict of interest.

## Publisher’s note

All claims expressed in this article are solely those of the authors and do not necessarily represent those of their affiliated organizations, or those of the publisher, the editors and the reviewers. Any product that may be evaluated in this article, or claim that may be made by its manufacturer, is not guaranteed or endorsed by the publisher.
